# Primary Localized Amyloidosis of Urinary Bladder: A Case Report and Review of Literature

**DOI:** 10.5812/numonthly.10870

**Published:** 2013-11-13

**Authors:** Kapeel Raja, Ejaz Ahmed, Muhammed Mubarak, Tanveer Iqbal, Syed Mujahid Hassan

**Affiliations:** 1 Department of Hepatogastroenterology, Sindh Institute of Urology and Transplantation, Karachi, Pakistan; 2 Department of Nephrology, Sindh Institute of Urology and Transplantation, Karachi, Pakistan; 3 Department of Histopathology, Sindh Institute of Urology and Transplantation, Karachi, Pakistan

**Keywords:** Amyloidosis, Urogenital System, Urinary Bladder

## Abstract

Amyloidosis is a disorder of protein metabolism characterized by extracellular deposition of abnormal protein fibrils. It may either be localized to any organ or systematically distributed throughout the body. The biochemical nature of proteins varies but the physical and tinctorial properties are shared by all the amyloidogenic proteins. In the West, it is mainly composed of amyloid light (AL) type immunoglobulin (Ig) light chains. Amyloidosis of the genitourinary tract is rare except for the kidney and isolated primary amyloidosis of the urinary bladder is even rarer. It mainly presents as intermittent painless gross hematuria. It mimics transitional cell carcinoma on imaging and endoscopic examination. We herein present a case of fifty six-years-old male with history of painless hematuria for three months. Cystoscopy revealed a 1 cm hyperemic area on the posterior wall of urinary bladder. The biopsy showed features of amyloidosis and amyloid A (AA) immunostaining was negative. Extensive workup was done to exclude other sites of involvement and a final diagnosis of primary localized amyloidosis of the urinary bladder was made. The patient is on regular follow-up.

## 1. Background

Amyloidosis is a disorder of protein metabolism characterized by extracellular deposition of abnormal protein fibrils (1-5). It may either be localized to any organ or systematically distributed throughout the body. Primary localized amyloidosis of the genitourinary tract is rare and similar involvement of the isolated urinary bladder is even rarer. The biochemical nature of amyloidogenic proteins varies, but all share similar physical and tinctorial properties. The nature of underlying protein disorder varies depending on racial and geographic factors (6-10). In the West, it is mainly amyloid light (AL) type immunoglobulin (Ig) light chain. Primary isolated amyloidosis of the urinary bladder mainly presents as intermittent painless gross hematuria (1, 3, 6). A few patients may present solely with irritative bladder symptoms. The onset of disease is often in the 6th to 8th decades of life similar to that of transitional cell carcinoma (7). It needs prompt evaluation in order to rule out systemic amyloidosis, which requires different management and has worse prognosis (1-5). We are presenting a case of primary localized amyloidosis of the urinary bladder with the best outcome.

## 2. Case Report 

**Figure 1.**
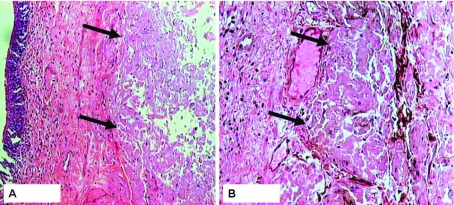
A. Hematoxylin and Eosin Staining of Bladder Biopsy Showing Nodular Deposits of Hyaline, a Cellular Material in the Lamina Propria of the Urinary Bladder (arrows). Overlying epidermis is unremarkable (H&E, ×200). B. Sliver staining showing negative staining of the hyaline material with this stain (arrows). A few scattered collagen fibers and basement membranes of capillaries are stained black. (GMS, ×200).

A fifty six-years-old male, doctor by profession, with no known co-morbid, presented with the history of hematuria for three months. Bleeding was painless, red in color, fresh, small in amount, intermittent, one to two episodes in a week, and occurred at the end of the micturition and it was not associated with any urgency, hesitancy, increased nocturnal frequency, fever, nausea or vomiting and abdominal pain. He gave no history of trauma or catheterization either recently or in the past. He denied any history of diabetes mellitus (DM), hypertension, and ischemic heart disease (IHD). He has family history of DM, hypertension and IHD for which he was taking some thrombolytic therapy (Aspirin) and lipid lowering drugs (Statins) for the prevention of IHD since one and half year, but abandoned six months back since first episode of painless hematuria. His general physical examination and systemic examination were unremarkable. His vitals were stable. On laboratory findings, his complete blood picture showed hemoglobin of 11.5 g/dL with normal red cell indices, white cell count and platelets. His liver function tests, renal function tests, electrolytes, and coagulation were within normal limits. His urinary microscopic examination showed normal pH and specific gravity, no proteins and ketones, numerous RBCs, few WBCs, no casts, or crystals. He underwent ultrasound kidney, ureter, and bladder (KUB), which revealed a 0.5 cm nodular growth in the posterior wall of urinary bladder and mild fullness in the left pelvicalyceal system. CT scan was performed, which showed a 0.3 cm non-obstructing calculus in the left pelvicalyceal system. He later underwent cystoscopic examination, which revealed a 1 cm hyperemic area on the posterior wall of the urinary bladder; four quadrant biopsies were taken. No other abnormality was detected. Out of four biopsy tissues, two revealed features of amyloidosis and AA was negative (Figure 1). The 24 hour urinary protein screening was negative for significant proteinuria. Urinary and serum protein immunofixation was negative. Skeletal survey was negative for lytic lesions. Rectal biopsy was negative for amyloidosis. Echocardiography and nerve conduction studies were performed to rule out amyloidosis, which were also negative. Free serum light chain assay was done, which showed a normal kappa/lambda ratio. Patient was discharged and advised routine follow up in urology outpatient department. Since 2 years he is asymptomatic. His serum free light chain was checked every six months, which has been within normal limits. His repeat ultrasound at six months was normal. His last annual cystoscopy revealed no gross growth or mucosal abnormality. 

## 3. Discussion

The amyloid term was historically coined by Virchow in 1854 (1). Solonis first described bladder amyloidosis in 1897 at autopsy (1). Primary amyloidosis of the urinary tract is a rare condition with less than 200 cases reported until the date (2). It is a disorder of protein metabolism characterized by extracellular deposition of abnormal fibrillary proteins. It may be localized or systematically distributed throughout the body (3). Our patient suffered from the localized amyloidosis of the urinary bladder. Amyloidosis of the bladder primarily affects posterior and lateral walls with unknown etiology. In our patient posterior wall was involved. It is mainly immunoglobulin light chain AL type (4). It mainly presents as intermittent painless gross hematuria. Our patient also presented with the similar complains. A few patients may present solely with irritative bladder symptoms. The onset of disease is often in the 6th to 8th decades of life similar to that of infiltrative carcinoma (4). It is a rare condition but recurrence is common. Amyloidosis of the bladder presents a great challenge to the urologist because of its close resemblance with an infiltrating neoplasm of bladder (5). Patients usually need early evaluation in order to rule out systemic amyloidosis, which requires different management and has poor prognosis (6). The association of amyloidosis with various malignancies, particularly multiple myeloma, medullary carcinoma of the thyroid, Hodgkin’s disease, and renal cell carcinoma has been well documented (7). Our patient underwent bone marrow examination and CT scan abdomen which were both normal. No evidence of associated disorder was found. Follow-up cystoscopy performed after one year it was also normal. Urinary dysfunction is found in approximately 50% of cases in patients due to amyloid neuropathy. Cystoscopic appearance varies from a solid circumscribed elevated sessile lesion to a grossly congested and/or ulcerated mucosa with petechial hemorrhages involving the whole urinary bladder. Typically, in localized amyloidosis of the bladder, amyloid deposits are demonstrated in the lamina propria and muscular is propria with vessel wall involvement. Similar findings were also seen in our patient (8). Some studies have found anti-inflammatory agents such as oral colchicine to be beneficial in the focal bladder amyloidosis while diffuse or locally extensive bladder involvement usually requires conventional transurethral resection that should be supplemented with intravesical dimethyl sulfoxide (DMSO) instillation with periodic cystoscopy (9, 10). As our patient was asymptomatic, no treatment was offered, but yearly cystoscopy was advised to rule out recurrence and progression of the primary disease.

## 4. Conclusions

In conclusion, primary localized urinary bladder amyloidosis is a rare disease. Life-long surveillance is mandatory. Endoscopic resection is the adequate treatment for focal disease. Intravesical DMSO and oral colchicine are beneficial adjunctive therapies to resection of the lesions, for more extensive involvement of the bladder.
